# Chloroquine Versus Dihydroartemisinin-Piperaquine With Standard High-dose Primaquine Given Either for 7 Days or 14 Days in *Plasmodium vivax* Malaria

**DOI:** 10.1093/cid/ciy735

**Published:** 2018-08-24

**Authors:** Cindy S Chu, Aung Pyae Phyo, Claudia Turner, Htun Htun Win, Naw Pet Poe, Widi Yotyingaphiram, Suradet Thinraow, Pornpimon Wilairisak, Rattanaporn Raksapraidee, Verena I Carrara, Moo Kho Paw, Jacher Wiladphaingern, Stéphane Proux, Germana Bancone, Kanlaya Sriprawat, Sue J Lee, Atthanee Jeeyapant, James Watson, Joel Tarning, Mallika Imwong, François Nosten, Nicholas J White

**Affiliations:** 1 Shoklo Malaria Research Unit, Mahidol–Oxford Tropical Medicine Research Unit, Faculty of Tropical Medicine, Mahidol University, Mae Sot, Thailand; 2 Centre for Tropical Medicine and Global Health, Nuffield Department of Medicine, University of Oxford, United Kingdom; 3 Mahidol–Oxford Tropical Medicine Research Unit; 4 Department of Molecular Tropical Medicine and Genetics, Faculty of Tropical Medicine, Mahidol University, Bangkok, Thailand

**Keywords:** *Plasmodium vivax*, radical cure, primaquine, chloroquine, dihydroartemisinin-piperaquine

## Abstract

**Background:**

Primaquine is necessary for the radical cure of *Plasmodium vivax* malaria, but the optimum duration of treatment and best partner drug are uncertain. A randomized controlled trial was performed to compare the tolerability and radical curative efficacy of 7-day versus 14-day high-dose primaquine regimens (total dose 7mg/kg) with either chloroquine or dihydroartemisinin-piperaquine.

**Methods:**

Patients with uncomplicated *P. vivax* malaria on the Thailand-Myanmar border were randomized to either chloroquine (25mg base/kg) or dihydroartemisinin-piperaquine (dihydroartemisinin 7mg/kg and piperaquine 55mg/kg) plus primaquine, either 0.5 mg/kg/day for 14 days or 1 mg/kg/day for 7 days. Adverse events within 42 days and 1-year recurrence rates were compared and their relationship with day 6 drug concentrations assessed.

**Results:**

Between February 2012 and July 2014, 680 patients were enrolled. *P. vivax* recurrences (all after day 35) occurred in 80/654 (12%) patients; there was no difference between treatments. Compared to the 7-day primaquine groups the pooled relative risk of recurrence in the 14-day groups was 1.15 (95% confidence interval 0.7 to 1.8). Hematocrit reductions were clinically insignificant except in G6PD female heterozygotes, 2 of whom had hematocrit reductions to <23% requiring blood transfusion.

**Conclusion:**

Radical cure should be deployed more widely. The radical curative efficacy in vivax malaria of 7-day high-dose primaquine is similar to the standard 14-day high-dose regimen. Chloroquine and dihydroartemisinin-piperaquine are both highly effective treatments of the blood stage infection. Quantitative point of care G6PD testing would ensure safe use of the 7-day high-dose primaquine regimen in G6PD heterozygous females.

**Clinical Trials Registration:**

NCT01640574.

## Teaser

Chloroquine or dihydroartemisinin-piperaquine with either 7- or 14-day primaquine regimens provided highly effective radical cure of vivax malaria on the Thailand-Myanmar border. Short course higher dose primaquine regimens for vivax malaria radical cure may improve efficacy by improving adherence.

*Plasmodium vivax* is the most geographically dispersed of the human malarias, once extending to the Arctic circle and now as far north as the Korean peninsula [1]. Chloroquine has been the first-line treatment for over 70 years [2]. In recent years low grade chloroquine resistance, manifested by earlier appearance of relapses, has been reported increasingly, but high grade resistance remains confined to Oceania and Indonesia [3]. Piperaquine (a slowly eliminated bisquinoline) combined with dihydroartemisinin is well tolerated and highly effective against chloroquine resistant vivax malaria and has replaced chloroquine as first-line treatment in Indonesia [4].

The majority of *P. vivax* infections in East Asia and Oceania result from relapses [5, 6]. These occur usually at 5–9 week intervals following slowly eliminated antimalarial treatments. Relapses are a major cause of morbidity, particularly in children. The only currently available antirelapse drug is the 8-aminoquinoline primaquine. The most widely used radical cure regimen is 0.25 mg base/kg/day over 14 days [2]. Some studies report poor adherence to the 14-day regimen [7–10]. Shorter 5-day regimens at lower total doses have been recommended in the past [11, 12], but they are ineffective [13, 14]. Nearly all South American countries now recommend a 7-day regimen with the same total dose (3.5mg/kg: adult dose 210 mg) as the 14-day regimen. This is considered to improve adherence and thus effectiveness. In Southeast Asia and Oceania higher doses (7mg base/kg divided over 14 days: adult dose 420 mg) are required for radical cure. Preliminary trial data suggest this total dose can also be given safely over 7 days [15, 16].

This open 2-way randomized controlled trial compared the radical curative efficacy of short course 7-day high-dose primaquine with the standard 14-day high-dose regimen. Patients were also randomized either to chloroquine or dihydroartemisinin-piperaquine for treatment of the blood stage *P. vivax* infection.

## Ethics Approval

This study was approved by both the Mahidol University Faculty of Tropical Medicine Ethics Committee (MUTM 2011–043, TMEC 11-008) and the Oxford Tropical Research Ethics Committee (OXTREC 17-11) and was registered at ClinicalTrials.gov (NCT01640574).

## METHODS

### Setting

This study was conducted by the Shoklo Malaria Research Unit (SMRU), which operates clinics along the Thailand-Myanmar border. This is an area of hill forest and low seasonal malaria transmission. The patient population comprised migrant workers and displaced persons of Burman and Karen ethnicities [17].

### Participants and Study Procedures

Patients ≥6 months and ≥7 kg with uncomplicated *P. vivax* monoinfections were included. Patients were excluded if glucose-6-phosphate dehydrogenase (G6PD) deficient by the fluorescent spot test (FST), were pregnant or breastfeeding an infant ≤ 6 months, had a hematocrit ≤25%, or received a blood transfusion within 3 months. Written informed consent was obtained from patients or from parents or guardians of children less than 18 years old.

### Randomization

Randomization was computer generated in blocks of 20. Patients were randomized to either:

1. Chloroquine (25 mg base/kg; Remedica, Ltd., Cyprus) over 3 days and primaquine (1 mg base/kg/day; Government Pharmaceutical Organization, Thailand) for 7 days: CP7
2. Chloroquine as above and primaquine (0.5 mg base/kg/day) for 14 days: CP14
3. Dihydroartemisinin-piperaquine (dihydroartemisinin 7 mg/kg and piperaquine 55 mg/kg; Guilin Pharmaceutical company, China) over 3 days and primaquine (1 mg base/kg/day) for 7 days: DP7
4. Dihydroartemisinin-piperaquine as above plus primaquine (0.5 mg base/kg/day) for 14 days: DP14

Food and drink were given before drug administration. All doses were supervised.

### Enrolment Procedures

At enrolment (day 0) a medical history and vital signs were recorded, and a physical examination was performed. A malaria smear, hematocrit, complete blood count (CBC), and urine β-human chorionic gonadotropin (β-hCG) pregnancy test were performed. Parasite cultures were taken when densities exceeded 300 per 500 white blood cells (WBC) (approximately 4500/µL). Three blood spots on filter paper (Whatman 3MM) were collected for human and parasite genotyping.

### Follow-up

Vital signs, concomitant medications, and adverse events were assessed at each follow-up visit. A malaria smear was taken daily until negative. Fever clearance was defined as the interval to an aural temperature <37.5° C on 2 consecutive daily measurements. A venous plasma blood sample for antimalarial drug levels was taken on day 6. Methemoglobin was measured using a transcutaneous pulse oximeter (Masimo® Radical-7) on days 0, 3, 6, and 13 and additionally on day 10 in the primaquine 14-day groups. Follow-up visits continued at weeks 2 and 4 and then every 4 weeks until 52 weeks. At each visit, a malaria smear and CBC were taken. Urine β-HCG pregnancy testing was performed in females of childbearing potential.

### Laboratory Investigations

Malaria blood films were stained with Giemsa, and parasites were counted per 1000 red cells or per 500 WBC. In vitro susceptibility assays were performed when >80% ring stages were present. Hematocrits were measured with a Hawksley Micro-Haematocrit reader. The CBC was performed using a CeltacF MEK-8222K hematology analyzer (Nihon Kohden, Japan). The G6PD FST (R&D Diagnostic, Greece) was performed as described previously [18]. Chloroquine/desethylchloroquine, piperaquine, and primaquine/ carboxyprimaquine blood concentrations were measured using 3 validated liquid chromatography (LC)—tandem mass spectrometry (MS/MS) methods. The lower limits of quantification (LLOQ) were set to 1.13, 1.20, 0.912 and 3.90 ng/mL for chloroquine/desethylchloroquine, piperaquine, primaquine, and carboxyprimaquine, respectively. All 3 quantification methods were validated according to regulatory standards, and 3 levels of quality control samples were analyzed in triplicate within each batch of clinical samples. Total imprecision (ie, relative standard deviation) for all quality control samples was below 10% during drug quantification.

### Malaria Infections Recurring During Follow-up

Enrolment study procedures were repeated for *P. vivax* recurrences. Blood was taken again for chloroquine or piperaquine levels. Standard high-dose primaquine (0.5 mg/kg/day for 14 days) and chloroquine were then given. Follow-up was restarted as if newly recruited. The total study duration remained 52 weeks from enrolment. For *P. falciparum* infections, standard treatment with mefloquine and artesunate or dihydroartemisinin-piperaquine [2] was given and follow-up continued without interruption.

### Pregnancy During Follow-up

Patients with a positive urine β-HCG pregnancy test during follow-up were referred to the antenatal clinic, and study follow-up was continued. If any malaria infection occurred during pregnancy, the patient was censored from the study and managed in the antenatal clinic.

### Adverse Events and Serious Adverse Events

Adverse event data were collected until day 42. Patients were treated with hematinics for hematocrit <30% or <34% if under 2 years old. The blood transfusion threshold was a hematocrit <18% or if the patient was symptomatic. Inpatient observation was performed for symptomatic methemoglobin elevation or if ≥15% without symptoms. Serious adverse events were reported to a Data Safety Monitoring Board within 24 hours and the local ethics committee within 7 days.

### Sample Size

Based on earlier experience at SMRU, the annual *P. vivax* recurrence rate in the 14-day groups was anticipated to be ~18%. With a 1-sided alpha of 0.025, power 80%, and up to 20% loss to follow-up, a sample size of 680 (170 subjects per arm) was necessary to show noninferiority of the 7-day primaquine groups with a delta of 10% compared to the 14-day groups [19, 20].

### Statistical Analysis

The primary endpoint was the cumulative risk of *P. vivax* recurrence by 52 weeks. Secondary endpoints included the cumulative risk of *P. vivax* recurrence by 8 weeks, adverse events within 42 days, and the relationship of day 6 drug concentrations with the risk of recurrence. A per protocol time-to-event (ie, time to first recurrence) analysis was performed using Cox proportional hazards model, with premature discontinuations right-censored. A second, mixed-effects Cox model was fitted to all data, with individual random effects for multiple recurrences. Both models included sex, treatment arm, and the log_10_ day primaquine and carboxy-primaquine concentrations as independent variables. A Cox proportional hazards model was also used to assess the risk factors associated with premature discontinuation. Groups were compared using Pearson χ^2^ test, nonparametric K-sample test, multivariable linear or logistic regression as appropriate. Thirty-four G6PD heterozygous females (determined by G6PD spectrophotometry and genotyping) were excluded from the hematocrit analyses (except for enrolment data). These data were reported previously [21]. Stata 15.1 (StataCorp, College Station, TX, USA) and R version 3.4.3 (open source) were used for the data analysis.

## RESULTS

### Patient Characteristics

**Figure 1.**
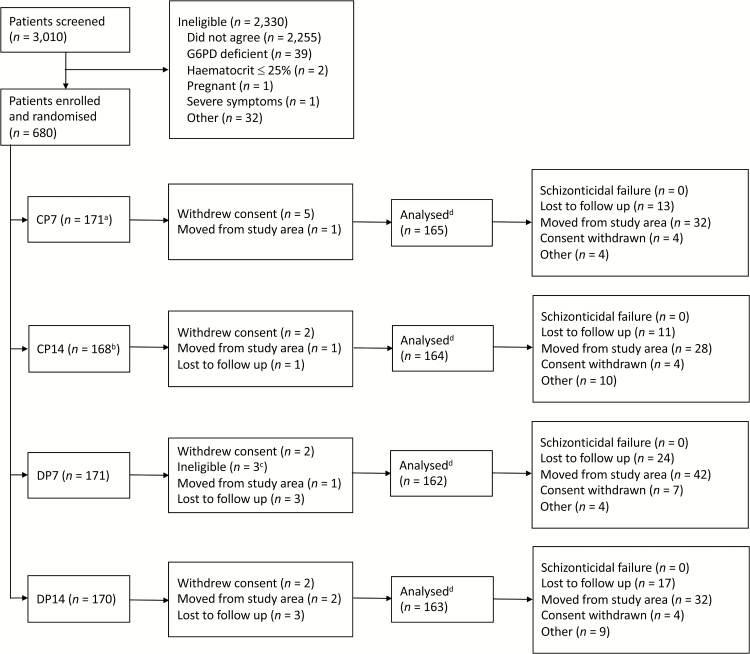
Study diagram. ^a^One patient randomized to the CP7 arm was given DP7 ^b^Two patients randomized to the CP14 arm were given CP7 ^c^Two patients vomited the drug treatment twice and one patient had received a blood transfusion in the prior 3 months. ^d^A total of 26 patient were excluded from the analyses because they had not completed the schizonticidal treatment. The three patients with incorrect randomization were included in the analysis. Abbreviations: CP7, Chloroquine + Primaquine 1 mg/kg/day for 7 days; CP14, Chloroquine + Primaquine 0.5 mg/kg/day for 14 days; DP7, Dihydroartemisinin-piperaquine + Primaquine 1 mg/kg/day for 7 days; DP14, Dihydroartemisinin-piperaquine + Primaquine 0.5 mg/kg/day for 14 days; G6PD, glucose-6-phosphate dehydrogenase.

Between February 2012 and July 2014, 680 patients with uncomplicated acute vivax malaria were enrolled (Figure 1). Patient characteristics were similar between treatment arms (Tables S1 and S2). Splenomegaly was more common in the 5–15 year age group (6%) (Supplementary Table S3). Mean (95% confidence interval [CI]) presenting hematocrit was higher in males (41% [40.2 to 41.1]) than females (37% [36.2 to 37.3]), and in the >15 year age group [(41% [40.4 to 41.3]) than the <5 year (33% [31.2 to 34.1]) and 5–15 year (37% [36.2 to 37.3]) groups (Supplementary Table S4).

### Premature Study Discontinuation

Of the 654 patients analyzed, 133 (20%) did not complete 6 months follow-up; a further 113 (17%) did not complete 1-year follow-up. In the multivariable Cox regression model, independent risk factors for premature discontinuation were being >15 years old (hazard ratio [HR] 2.6 [95% CI 1.1 to 6.3], comparator 0–4 years old; *P* = .037), being male (HR 1.6 [95% CI 1.2 to 2.1]; *P* = .002), and taking dihydroartemisinin-piperaquine (HR 1.4 [95% CI 1.1 to 1.8]; *P* = .009). Moving out of the study area (199/246; 81%) was the most common reason for premature discontinuation.

### Treatment Efficacy

Fever resolution was faster in the dihydroartemisinin-piperaquine groups [mean difference 0.5 days (95% CI 0.31 to 0.58); *P* < .001). Median days (interquartile range [IQR], range) to fever clearance were CP7 (1 [1 to 2, 1 to 4]), CP14 (2 [1 to 2, 1 to 4]), DP7 (1 [1 to 2, 1 to 4]), and DP14 (1 [1 to 1, 1 to 2]). Over 95% of the patients cleared fever by day 2 in all arms except the CP7 arm (Supplementary Table S5). Parasite clearance was also faster in the dihydroartemisinin-piperaquine groups (mean difference 0.7 days [95% CI 0.61 to 0.79]; *P* < .001). Median days (IQR, range) to parasite clearance were CP7 (2 [2 to 2, 1 to 4]), CP14 (2 [2 to 3, 1 to 5]), DP7 (1 [1 to 2, 1 to 4]), and DP14 (1 [1 to 2, 1 to 3]). By day 3, over 95% of patients were aparasitemic (Supplementary Table S6).

### Post-treatment Suppression of Recurrent Infections

**Figure 2.**
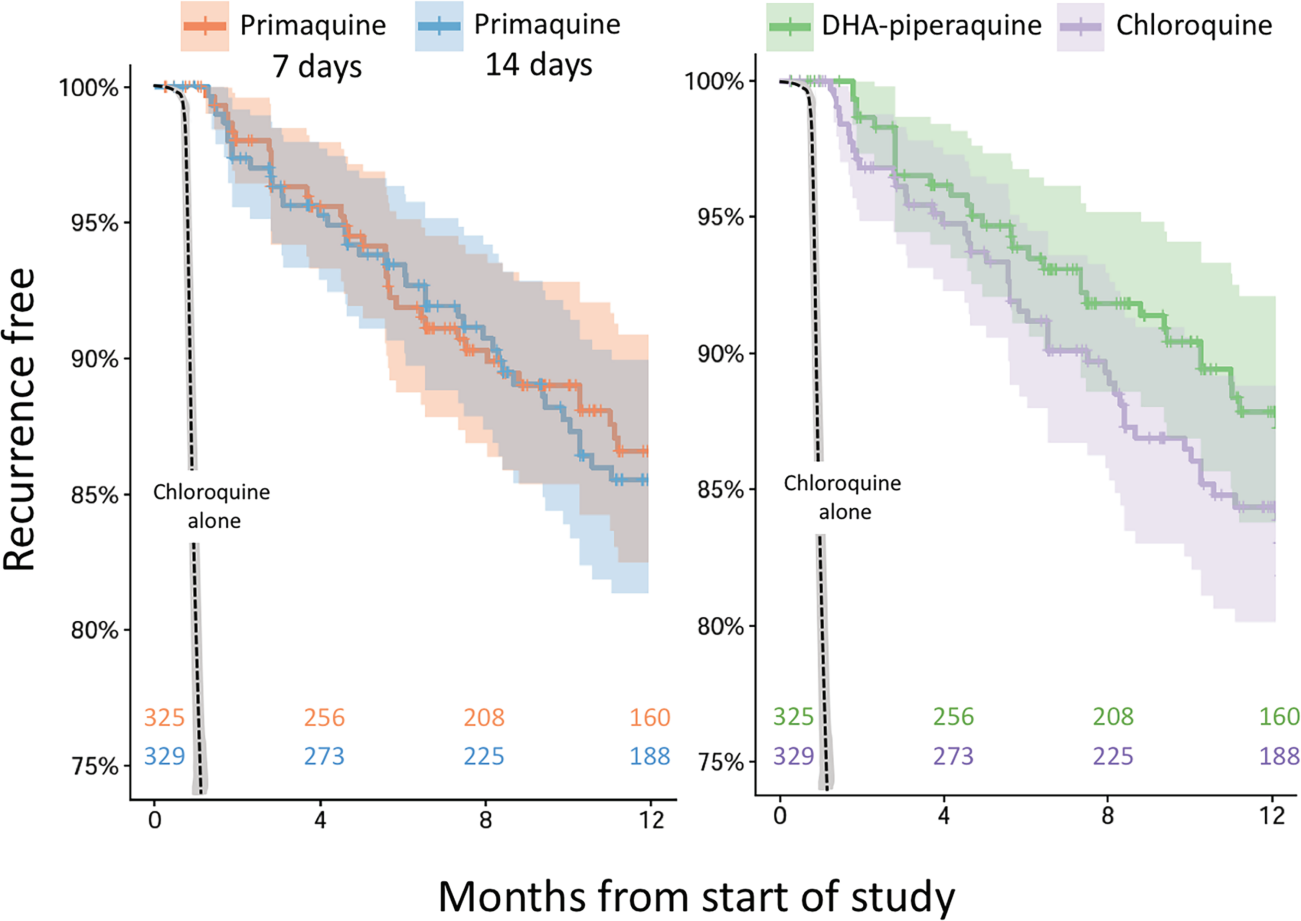
Recurrence of *Plasmodium vivax* malaria during the 1-year study. Survival curves comparing marginal time to first recurrence in the 2 schizontocide groups (*left panel*) and the 2 radical cure groups (*right panel*). Vertical notches denote right censored observations. 95% confidence intervals are shown by the shaded areas. The dashed black line shows the proportion recurring when not given radical cure in recent (two years previous) historical controls from the same study site [22].

At this location, the majority of *P. vivax* recurrences (mainly relapses) without primaquine occur within 8 weeks, and over 90% occur within 4 months [22] (Figure 2). In this study the earliest recurrence was on day 37. First recurrences were slightly earlier in the chloroquine groups: between weeks 6 and 8 there were 10 recurrences of 361 at risk in the chloroquine arms, and 4 of 326 in the piperaquine arm (all in week 8) (Figure 2).

### Radical Curative Efficacy

**Figure 3.**
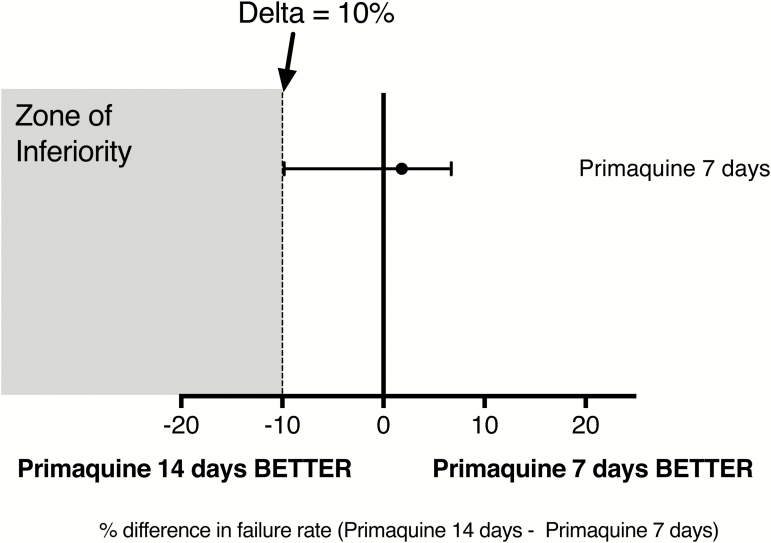
Noninferiority analysis of the 7-day versus 14-day high-dose primaquine regimens. The relative risk of a new *Plasmodium vivax* infection in the 7-day high-dose primaquine regimen (13.5%) was not inferior to the 14-day regimen (15.3%). The difference in relative risk between the two regimens was 1.8% (95% confidence interval −9.8 to +6.7%).

*P. vivax* recurrences occurred in 80/654 (12%) patients: an overall rate of 0.16 infections/ person-year. This rate was similar between arms (Figure 2 and Table 1). The 4-month radical curative efficacy of primaquine was > 95% in all arms; *P* = .548. Median (range) intervals to first recurrence were chloroquine group 168 (37 to 368) days, dihydroartemisinin-piperaquine group 170 (53 to 370) days, primaquine 14-day group 196 (39 to 370) days, and primaquine 7-day group 167 (37 to 368) days. The estimated first recurrence rate at 52 weeks (95% CI) after 7-day primaquine (13.5% [9.3 to 17.6]) was noninferior to 14-day primaquine (15.3% [10.9 to 19.5]) (Figure 3). This corresponded to a pooled relative risk of recurrence (95% CI) in the 14-day compared to the 7-day primaquine groups of 1.15 (0.7 to 1.8). The pooled relative risk of the chloroquine groups was 1.3 (0.8 to 2) compared to dihydroartemisinin-piperaquine. Patients 5–15 years old had a higher risk of early recurrence (odds ration [OR] 5.4 [95% CI 1.6 to 17.5]) than adults > 15 years old. Subsequent recurrences after retreatment with standard CP14 occurred in 10/80 (13%) patients.

**Table 1.** Rates of First *Plasmodium vivax* Infections Within 1 Year

Treatment Group^a^	First *P. vivax* Infection Within 1 Year, *n* (%)	Person-Time (Years)	Rate of New Infection	95% CI	*P*-Value^b^
CP7 (n = 165)	20 (12%)	128	0.16	0.10 to 0.24	.256
CP14 (n = 164)	26 (16%)	125	0.22	0.15 to 0.32	Comparator
DP7 (n = 162)	17 (11%)	114	0.15	0.09 to 0.24	.247
DP14 (n = 163)	16 (10%)	122	0.13	0.08 to 0.22	.119
All groups	80 (12%)	488	0.16	0.13 to 0.21	-

Abbreviation: CI, confidence interval.

^a^CP7: Chloroquine + Primaquine 1 mg/kg/day for 7 days.

CP14: Chloroquine + Primaquine 0.5 mg/kg/day for 14 days.

DP7: Dihydroartemisinin- piperaquine + Primaquine 1 mg/kg/day for 7 days.

DP14: Dihydroartemisinin-piperaquine + Primaquine 0.5 mg/kg/day for 14 days.

^b^A Cox proportional hazards model was used to compare differences in the rate of first new infection between treatment groups.

### *Plasmodium falciparum* Infections

There were 25/654 (4%) *P. falciparum* infections during the 1 year follow-up. The incidence of *P. falciparum* infections was not different either between the pooled dihydroartemisinin-piperaquine and chloroquine groups: 10/325 (3.1%) versus 15/329 (4.6%); *P* = .378, or between the pooled 7-day and 14-day primaquine groups: 8/327 (2.5%) versus 17/327 (5.2%); *P* = .073.

### Radical Cure Effectiveness and the Relationship With In Vivo Drug Concentrations and In Vitro Susceptibility

**Figure 4.**
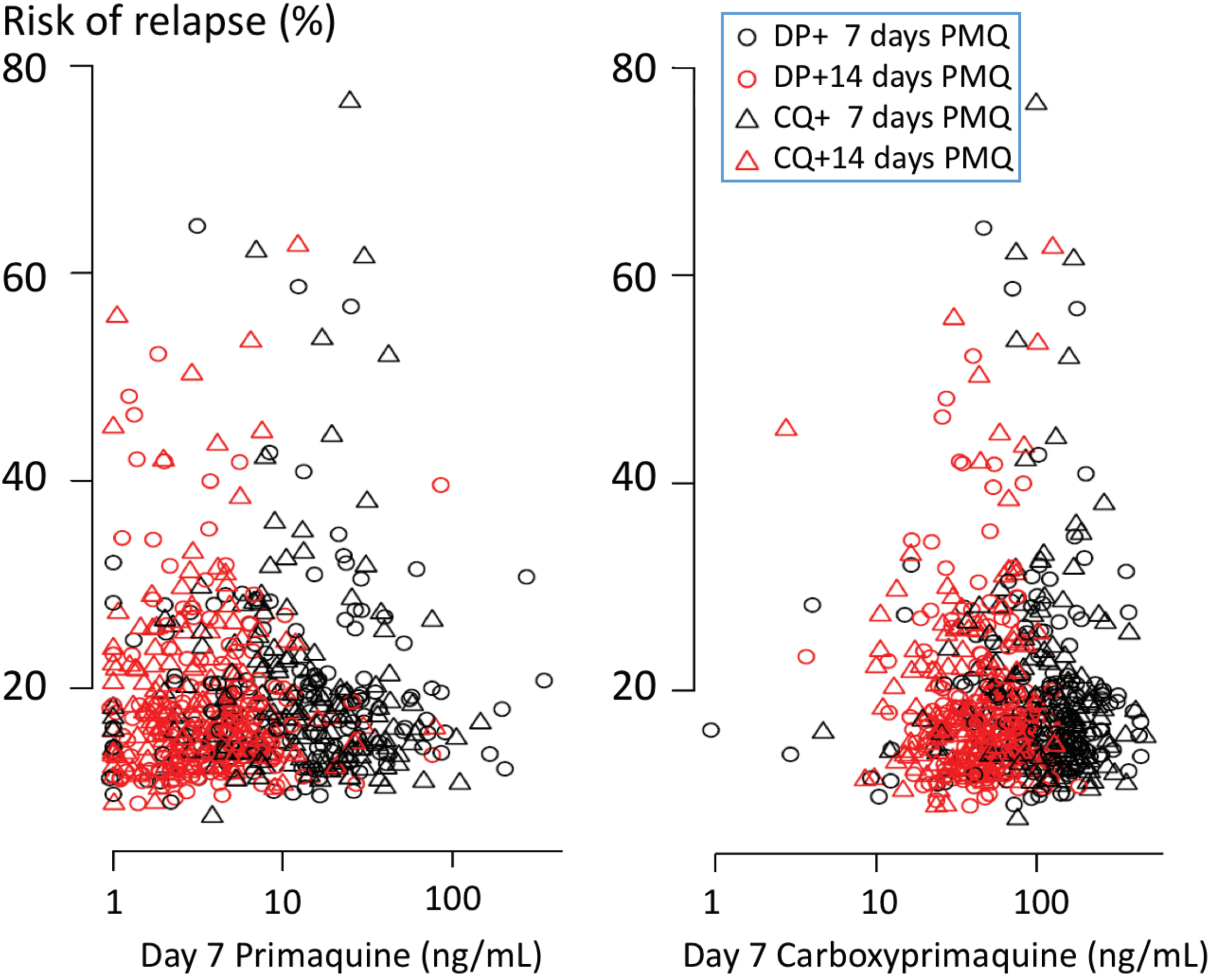
Risk of *Plasmodium vivax* relapse in relation to primaquine and carboxyprimaquine drug levels. The predicted individual risks of recurrence within 1 year as a function of day 7 primaquine (*left panel*) and carboxy-primaquine (*right panel*) concentrations. These relationships are estimated from a mixed effects Cox proportional hazards model where sex, schizonticide treatment, and primaquine and carboxy-primaquine concentrations were independent predictive covariates.

The majority of patients completed their primaquine treatment (Supplementary Table S7). There was no association between the day 6 total chloroquine or piperaquine concentrations and the interval to first *P. vivax* recurrence within 8 weeks. No patients had “therapeutic” blood chloroquine (≥10 ng/mL [23]) or piperaquine (≥30 ng/mL [24]) concentrations at week 8. In both the Cox proportional hazards model of time to first recurrence and the mixed-effects Cox model (all recurrences) higher day 6 concentrations of carboxyprimaquine (*P* = .04, both models) but not primaquine were associated with a lower risk of subsequent recurrence (Figure 4). The geometric mean (95% CI) of chloroquine IC_50_ in the 36 successful in vitro tests was 17.9 ng/mL (11.5 to 27.7). Six isolates (16.7%) had an IC_50_ > 50 ng/mL, suggesting resistance.

### Adverse Events

**Figure 5.**
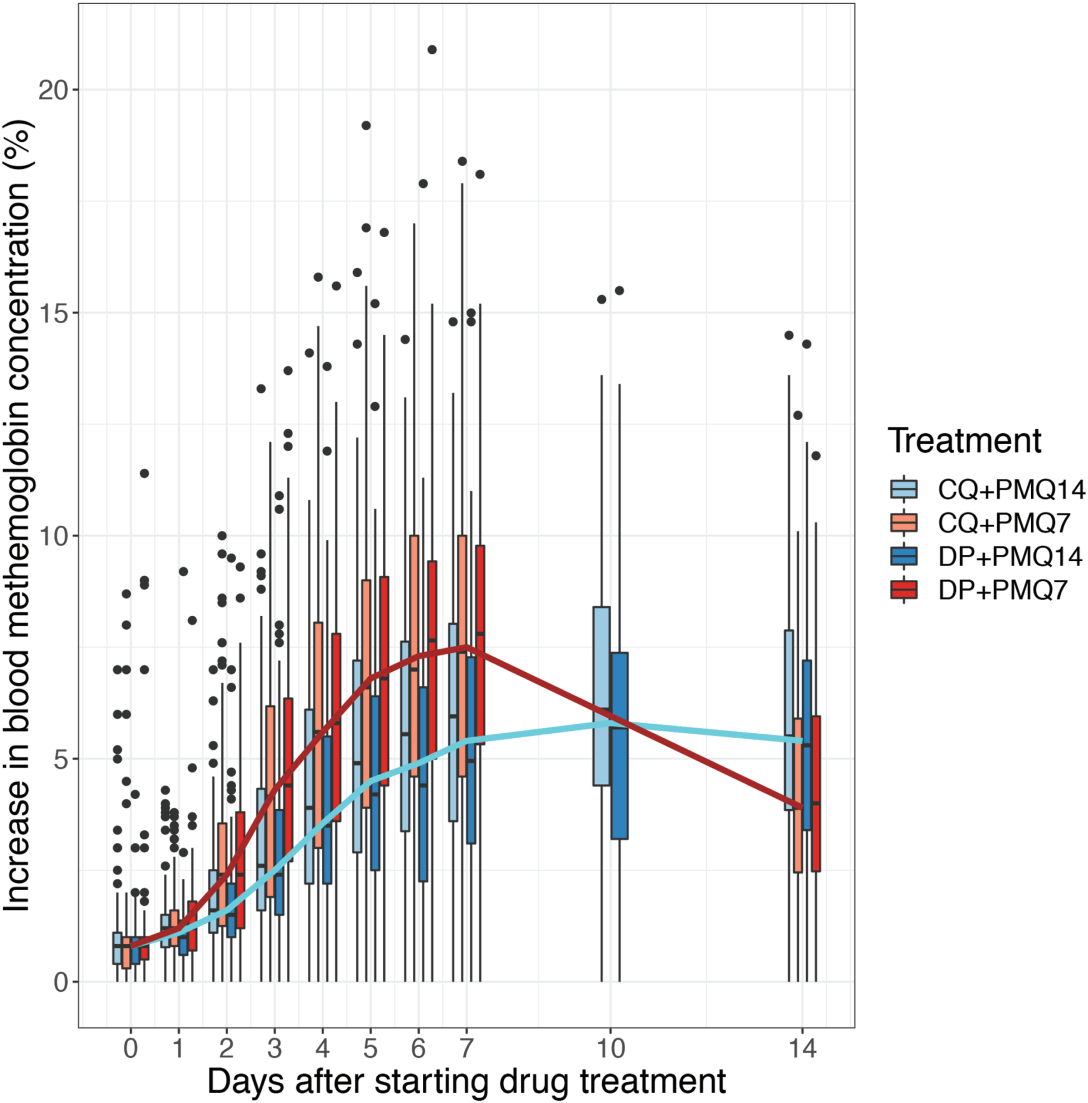
Median methemoglobin measurements during primaquine administration. Evolution of methemoglobin concentrations over time within the four study groups. Data from PMQ7 arm are shown in light and dark red for CQ and DP, respectively. Data from PMQ14 are shown in light and dark blue for CQ and DP, respectively. The thick brown and aqua lines correspond to the median trends for the pooled 7-day and 14-day primaquine groups, respectively. Abbreviations: CQ, chloroquine; DP, dihydroartemisinin-piperaquine, PMQ7, primaquine 1 mg/kg/day for 7 days; PMQ14, primaquine 0.5 mg/kg/day for 14 days.

Except for abdominal pain, there were no significant differences in reported adverse events between the 4 groups (Table 2). When pooled by schizonticide treatment, more abdominal pain was reported in the chloroquine 79/329 (24%) compared to the dihydroartemisinin-piperaquine group 38/325 (12%): *P* < .001. When pooled by primaquine treatment, more abdominal pain was reported in the 7-day 69/325 (21%) than the 14-day group 49/329 (15%):*P* = .032. As reported previously [19], hemolysis was substantially greater in 33 G6PD Mahidol female heterozygotes compared to the 241 homozygous wild-type normal females. In heterozygotes, the mean (95% CI) fractional reduction in hematocrit was 20% (14.8 to 26.0) in the 7-day group versus 13% (8.6 to 17.6) in the 14-day group; *P* = .039. Hematocrit fell below 20% (~7 g/dL hemoglobin) in one heterozygous female in the DP7 group (requiring blood transfusion). Another heterozygote female in the DP7 group received a blood transfusion for symptomatic hemolysis. In G6PD wild-type females the mean (95% CI) absolute hematocrit change at day 6 was similar between the 7-day (−0.5% [−1.2 to 0.8]) and 14-day primaquine groups (−0.5% [−1.2 to 0.3]). The same was true for male patients: (−1.0% [−1.5 to −0.5]) and (−0.6% [−1.1 to −0.1]), respectively. The peak transcutaneous methemoglobin measurements were higher in the 7-day primaquine groups: mean (95% CI) difference 1.8% (1.7 to 1.9); *P* < .001 (Figure 5 and Supplementary Table S8). Peri-oral cyanosis was noted in 17 patients. Primaquine was stopped in 5 patients (29%) because of associated symptoms (Supplementary Table S8). All resolved without further sequelae, and none had a subsequent *P. vivax* infection. There were 30 serious adverse events reported; most common were methaemoglobinaemia (n = 10), haemolysis (n = 3), and presumed bacterial infection (n = 10). Four deaths occurred. None was considered related to the study drugs.

**Table 2.** Adverse Events Occurring by Day 42

Adverse Event	CP7 *n* = 165	CP14 *n* = 164	DP7 *n* = 162	DP14 *n* = 163	*P*-Value^a^
Abdominal pain, *n* (%)	44 (33%)	35 (27%)	25 (20%)	13 (10%)	.001^b^
Anemia, *n* (%)	12 (7%)	17 (10%)	20 (12%)	18 (11%)	.479
Nausea or vomiting, *n* (%)	12 (7%)	10 (6%)	5 (3%)	9 (6%)	.404
Dizziness, *n* (%)	15 (9%)	22 (13%)	18 (11%)	29 (18%)	.106
Headache, *n* (%)	15 (9%)	15 (9%)	18 (11%)	21 (13%)	.635
Fatigue, *n* (%)	11 (7%)	9 (6%)	6 (4%)	10 (6%)	.668

^a^χ^2^ used for significance testing unless otherwise noted.

^b^Abdominal pain was significantly less in the DP14 arm compared to the other arms; logistic regression was used to compare differences between groups.

## DISCUSSION

Relapse of vivax malaria along the Thailand-Myanmar border was very effectively prevented by both the conventional 14-day high-dose regimen and the 7-day high-dose primaquine regimen. This is consistent with earlier studies with shorter follow-up periods [15, 16, 25, 26]. Only 14 (2%) of 654 patients had recurrent *P. vivax* infections within 8 weeks, whereas without primaquine, approximately 50% of patients experience relapse within 8 weeks of chloroquine treatment [27, 28] in this area (Figure 2). Without primaquine, 90% of all recurrences (mainly relapses) occur within 4 months [22]. However, with radical cure in this study, recurrences showed no periodicity, so they were most likely newly acquired infections after the post-treatment prophylaxis provided by chloroquine or piperaquine had waned.

Radical cure efficacy is thought to depend mainly on the total exposure to the bioactive metabolites of primaquine. In this study, higher day 6 concentrations of the more slowly eliminated biologically inactive metabolite carboxyprimaquine were associated with lower recurrence rates (Figure 4). This may reflect greater exposure to the active metabolites. The health benefits of preventing relapse are substantial. Where supervised treatment is not possible, the 7-day high-dose regimen should have an adherence advantage while providing similar radical curative efficacy to the standard 14-day regimen. But it is slightly less well tolerated with a higher incidence of abdominal discomfort and greater methemoglobinemia. More concerning was clinically significant hemolytic anemia in G6PD Mahidol female heterozygotes [21]. This is potentially a major limitation in areas where G6PD deficiency is prevalent and quantitative G6PD assessments are unavailable. Otherwise, the hematological consequences were mild. Although G6PD testing is recommended when radical cure is provided, it is usually unavailable in most endemic areas. Current qualitative tests identify only a minority of female heterozygotes. Higher daily primaquine dose regimens carry higher hemolytic risks. Quantitative G6PD tests are being developed, which would identify the vulnerable heterozygous females [29] allowing the 7-day high-dose primaquine regimen to be used safely. Further studies are in progress to assess the safety and effectiveness of the 7-day high-dose primaquine regimen in areas where other G6PD deficiency variants are prevalent [30].

Low grade chloroquine resistance in *P. vivax* is prevalent along the Thailand-Myanmar border, as evidenced by recent slowing of fever and parasite clearance, an increasing proportion of recurrences within 28 days of starting chloroquine alone and declining in vitro susceptibility [4]. Despite this, combination treatment with primaquine, which also has significant asexual stage activity against *P. vivax* [31], remains efficacious. As reported previously, dihydroartemisinin-piperaquine is a well-tolerated and highly efficacious alternative to chloroquine, which can be considered as first-line treatment, thereby providing a unified treatment for all malarias [2]. Dihydroartemisinin-piperaquine has the advantage of giving faster therapeutic responses (attributed to the artemisinin component), with shorter times to fever clearance, and slightly longer period of post-treatment suppression of recurrent infections. There was also a trend to lower recurrence rates with dihydroartemisinin-piperaquine (Figure 2), but this was not statistically significant.

A limitation of this study was the high rate of premature discontinuation, which was predominantly due to migration. These patients may be at higher risk for *P. vivax* malaria, so recurrence rates in this study could be underestimated.

In this region, both chloroquine and dihydroartemisinin-piperaquine combined with primaquine (given over 14 days or in the same total dose over 7 days) provide highly effective radical cure regimens in *P. vivax* malaria. Radical cure needs to be deployed more widely. Quantitative G6PD testing would ensure the safe use of 7-day high-dose primaquine regimens in G6PD heterozygous females.

## Supplementary Data

Supplementary materials are available at *Clinical Infectious Diseases* online. Consisting of data provided by the authors to benefit the reader, the posted materials are not copyedited and are the sole responsibility of the authors, so questions or comments should be addressed to the corresponding author.

Supplementary Tables

## Notes

***Acknowledgments.*** The authors thank the Shoklo Malaria Research Unit staff for their commitment and diligence in running this study with special appreciation for the work of Nay Lin Soe, Htet Htet Chaw, Nwe Wah Lin, Thida Zin, Say Paw, Naw Eh Shee, Thu Lay Paw, Patrick and the data entry team. We would also like to thank the Data Safety and Monitoring Board: Bob Taylor, Charles Woodrow, and Professor Nicholas Day for their time and commitment.

***Funding.*** This work was supported by the Wellcome Trust [grant number 089179/Z/09/Z] to N. J. W. C. S. C., G. B., F. H. N., and N. J. W. were supported by the Wellcome Trust [programme grant number 089179/Z/09/Z]. C. S. C., C. .T, G. B., S. J. L., A. J., J. W., J. T., F. N., and N. J. W. are part of the Wellcome Trust Mahidol University Oxford Tropical Medicine Research Programme funded by the Wellcome Trust.

***Potential conflicts of interest.*** All authors: No reported conflicts of interest. All authors have submitted the ICMJE Form for Disclosure of Potential Conflicts of Interest. Conflicts that the editors consider relevant to the content of the manuscript have been disclosed.
