# Supplementary material for: Chloroquine Versus Dihydroartemisinin-Piperaquine With Standard High-dose Primaquine Given Either for 7 Days or 14 Days in Plasmodium vivax Malaria
Source: Clin Infect Dis. 2018 Aug 24;68(8):1311–9. doi: 10.1093/cid/ciy735 (PMC6452005; doi:10.1093/cid/ciy735)
Supplement: Supplementary Tables [file ciy735_suppl_supplementary_tables.docx]

Supplement table 1. Patient demographics and histories

| Patient characteristic | CP7^a^ | CP14^a^ | DP7^a^ | DP14^a^ |
| --- | --- | --- | --- | --- |
| Total analyzed, *n* | 165 | 164 | 162 | 163 |
| Male, n (%) | 110 (67%) | 106 (65%) | 101 (62%) | 104 (64%) |
| Age in years,  median (IQR, range) | 19 (13-34, 3-60) | 20 (12.5-32, 1.4-61) | 20 (13-31, 2-63) | 21 (14-35, 1.5-63) |
| 0 - 4 years, *n* (%) | 5 (3%) | 6 (4%) | 7 (4%) | 7 (4%) |
| 5 - 15 years, *n* (%) | 54 (33%) | 53 (32%) | 53 (33%) | 41 (25%) |
| > 15 years, *n* (%) | 106 (64%) | 105 (64%) | 102 (63%) | 115 (71%) |
| Ethnicity |  |  |  |  |
| Burman, *n* (%) | 51 (31%) | 54 (33%) | 58 (36%) | 58 (36%) |
| Karen, *n* (%) | 103 (62%) | 99 (60%) | 92 (57%) | 95 (58%) |
| Years living along border,  median (IQR, range)^b^ | 5 (3-9, 0-32) | 5 (2-8, 0.1-38) | 4 (2-8, 0.1-22) | 5 (2-10, 0-31) |
| Attended school, *n* (%) | 120 (73%) | 120 (73%) | 119 (74%)^1^ | 111 (68%) |
| Location of occupation |  |  |  |  |
| Farm, *n* (%) | 77 (47%) | 89 (54%) | 74 (46%)^1^ | 70 (43%) |
| Forest, *n* (%) | 18 (11%) | 19 (12%) | 26 (16%)^1^ | 22 (14%) |
| Village, *n* (%) | 63 (38%) | 48 (29%) | 55 (34%)^1^ | 61 (37%) |
| Other, *n* (%) | 7 (4%) | 8 (5%) | 6 (4%)^1^ | 10 (6%) |
| History of fever, *n* (%) | 160 (97%) | 164 (100%) | 161 (99%) | 162 (99%) |
| Days of fever reported,  median (IQR, range) | 2 (2-3, 0-10) | 3 (2-3, 1-10) | 3 (2-3, 0-15) | 3 (2-3, 0-7) |
| History of chills, *n* (%) | 129 (78%) | 126 (77%) | 127 (78%) | 116 (71%) |
| History of headache, *n* (%) | 151 (93%)^3^ | 144 (89%)^3^ | 149 (94%)^3^ | 148 (92%)^2^ |
| History of cough, *n* (%) | 54 (32%) | 39 (24%) | 49 (30%) | 52 (32%) |
| History of difficulty breathing, *n* (%) | 7 (4%) | 9 (6%) | 9 (6%) | 8 (5%) |
| History of abdominal pain, *n* (%) | 37 (22%) | 37 (23%)^1^ | 28 (18%)^2^ | 34 (21%)^1^ |

^a^ CP7: Chloroquine + Primaquine 1 mg/kg/day for 7 days,

CP14: Chloroquine + Primaquine 0.5 mg/kg/day for 14 days,

DP7: Dihydroartemisinin- piperaquine + Primaquine 1 mg/kg/day for 7 days,

DP14: Dihydroartemisinin-piperaquine + Primaquine 0.5 mg/kg/day for 14 days

Numeric superscript represents missing or unknown data.

^b^ If the subject lived along the border < 1 month, this was entered as 0.

Supplement table 2. Patient characteristics at presentation

| Drug treatment^a^ | CP7 | CP14 | DP7 | DP14 |
| --- | --- | --- | --- | --- |
| Total analyzed, *n* | 165 | 164 | 162 | 163 |
| Temperature °C,  median (IQR, range) | 37.4 (36.8-38.5, 36-40.9) | 37.4 (36.9-38.2, 36-40.5) | 37.4 (36.9-38.1, 36-40.5) | 37.2 (36.6-38.2, 36-40.2) |
| Temperature >=37.5 °C, *n* (%)^b^ | 82 (50%) | 79 (48%) | 77 (48%) | 68 (42%) |
| Heart rate (beats/min), median (IQR, range) | 87 (80-100, 52-160) | 88 (80-100, 60-128) | 87 (80-100, 54-148)^2^ | 84 (79-96, 60-130) |
| Respiratory rate (breaths/min), median (IQR, range) | 24 (22-26, 18-48) | 24 (22-26, 12-40) | 24 (22-26, 18-40)^1^ | 24 (22-26, 16-36) |
| Systolic blood pressure (mmHg), median (IQR, range)^c^ | 100 (90-110, 80-140) | 100 (90-110, 70-140) | 100 (100-110, 80-190) | 100 (100-110, 80-160) |
| Diastolic blood pressure (mmHg), median (IQR, range)^c^ | 60 (60-70, 40-100) | 60 (60-70, 40-90) | 60 (60-70, 40-100) | 70 (60-70, 50-110) |
| Body Mass Index (kg/m^2)^, median (IQR, range) | 19 (16-21, 13-30)^1^ | 19 (16-21, 13-29) | 19 (16-21, 12-36)^1^ | 19 (17-21, 12-28) |
| Hepatomegaly, *n* (%) | 10 (6%) | 13 (8%) | 10 (6%) | 4 (3%) |
| Splenomegaly, *n* (%) | 4 (2%) | 3 (2%) | 4 (3%) | 3 (2%) |
| Parasitemia/µL,  geometric mean (95% CI) | 4004 (3188-5027) | 4270 (3404-5356) | 3931 (3105-4978) | 3533 (2785-4483) |
| Gametocytemia, *n* (%) | 130 (83%) | 137 (86%) | 128 (82%) | 129 (82%) |
| Field hematocrit %, mean (SD) | 39 (5.1) | 40 (5.6) | 39 (5.0) | 39 (5.0) |
| Laboratory hemoglobin g/dL, mean (SD) | 12.6 (1.6)^1^ | 12.7 (1.7)^2^ | 12.5 (1.7)^1^ | 12.6 (1.6)^1^ |
| White blood count x10^3^/µL, mean (SD) | 6.2 (1.9)^1^ | 6.2 (1.7)^2^ | 6.4 (2.2)^1^ | 6.2 (2.1)^1^ |
| Platelet count x10^3^/µL, median (IQR, range) | 131 (93-182, 24-422)^1^ | 128 (90-180, 20-429)^2^ | 132 (88-180, 29-418)^1^ | 134 (91-178, 37-521)^1^ |

^a^ CP7: Chloroquine + Primaquine 1 mg/kg/day for 7 days,

CP14: Chloroquine + Primaquine 0.5 mg/kg/day for 14 days,

DP7: Dihydroartemisinin- piperaquine + Primaquine 1 mg/kg/day for 7 days,

DP14: Dihydroartemisinin-piperaquine + Primaquine 0.5 mg/kg/day for 14 days

^b^ This value represents the temperature at enrolment and does not include patients who developed a fever within one day of enrolment (approximately 24 hours)

^c^ Blood pressure was performed in patients ≥ 12 years old (*n* = 526)

Numeric superscript represents missing or unknown data

Supplement table 3. Comparison of the prevalence of hepatomegaly and splenomegaly between age groups at enrolment

| Presenting examination | *n* (%) | Odds ratio | 95% CI | *p*-value |
| --- | --- | --- | --- | --- |
| Hepatomegaly |  |  |  |  |
| < 5 years | 4 (16%) | 5.6 | 1.71 to 18.6 | 0.005 |
| 5 - 15 years | 19 (10%) | 3.1 | 1.52 to 6.30 | 0.002 |
| > 15 years | 14 (3%) | Comparator | Comparator | Comparator |
| Splenomegaly |  |  |  |  |
| < 5 years | 0 | - | - | - |
| 5 - 15 years | 11 (6%) | 8.2 | 2.26 to 29.7 | 0.001 |
| > 15 years | 3 (1%) | Comparator | Comparator | Comparator |

Supplement table 4. Comparison of absolute hematocrit and platelet count at enrolment, by sex and by age group

|  | Mean | 95% CI | *p*-value |
| --- | --- | --- | --- |
| Field hematocrit (%) |  |  |  |
| Male | 41 | 40.2 to 41.1 | <0.001 |
| Female | 37 | 36.2 to 37.3 |  |
|  |  |  |  |
| < 5 years | 33 | 31.2 to 34.1 | <0.001 |
| 5 - 15 years | 37 | 36.2 to 37.3 | <0.001 |
| > 15 years | 41 | 40.4 to 41.3 | Comparator |
|  |  |  |  |
| Platelet count (x10^3^/µL) |  |  |  |
| Male | 133 | 126 to 139 | <0.001 |
| Female | 159 | 150 to 169 |  |
|  |  |  |  |
| < 5 years | 193 | 148 to 237 | <0.001 |
| 5 - 15 years | 150 | 139 to 161 | 0.013 |
| > 15 years | 135 | 129 to 141 | Comparator |

Supplement table 5. Proportion of patients with fever clearance by days 1 and 2^a^

|  | Day 1 | | Day 2 | | *p*-value^b^ |
| --- | --- | --- | --- | --- | --- |
| Treatment group^a^ | n (%) | 95 % CI | n (%) | 95 % CI |  |
| CP7 | 58 (65%) | 0.54 to 0.75 | 82 (92%) | 0.85 to 0.97 | 0.087 |
| CP14 | 44 (50%) | 0.39 to 0.60 | 86 (97%) | 0.91 to 0.99 | Comparator |
| DP7 | 63 (81%) | 0.70 to 0.89 | 76 (97%) | 0.91 to 0.99 | <0.001 |
| DP14 | 56 (82%) | 0.71 to 0.91 | 68 (100%) | 0.95 to 1 | <0.001 |

^a^ Patients with a temperature ≥37.5°C within one day of enrolment (approximately 24 hours) are included

^b^ CP7: Chloroquine + Primaquine 1 mg/kg/day for 7 days,

CP14: Chloroquine + Primaquine 0.5 mg/kg/day for 14 days,

DP7: Dihydroartemisinin- piperaquine + Primaquine 1 mg/kg/day for 7 days,

DP14: Dihydroartemisinin-piperaquine + Primaquine 0.5 mg/kg/day for 14 days

^c^ Ordered logistic regression used to compare the proportion of patients with fever clearance at each successive day

Supplement table 6. Proportion of patients with parasite clearance by days 1, 2 and 3

| Treatment group^a^ | Day 1 | | Day 2 | | Day 3 | | *p*-value^b^ |
| --- | --- | --- | --- | --- | --- | --- | --- |
|  | *n* (%) | 95 % CI | *n* (%) | 95 % CI | *n* (%) | 95 % CI |  |
| CP7 (*n*=165) | 26 (16%) | 10.6 to 22.2 | 146 (89%) | 82.6 to 92.9 | 164 (99%) | 96.7 to 99.9 | 0.082 |
| CP14 (*n*=164) | 30 (18%) | 12.7 to 25.1 | 123 (75%) | 67.7 to 81.4 | 158 (96%) | 92.2 to 98.7 | Comparator |
| DP7 (*n*=162) | 116 (72%) | 64.0 to 78.4 | 158 (99%) | 93.8 to 99.3 | 161 (99%) | 96.6 to 99.9 | <0.001 |
| DP14 (*n*=163) | 111 (68%) | 60.4 to 75.2 | 161 (99%) | 95.6 to 99.9 | 163 (100%) | 97.8 to 100 | <0.001 |

^a^ CP7: Chloroquine + Primaquine 1 mg/kg/day for 7 days,

CP14: Chloroquine + Primaquine 0.5 mg/kg/day for 14 days,

DP7: Dihydroartemisinin- piperaquine + Primaquine 1 mg/kg/day for 7 days,

DP14: Dihydroartemisinin-piperaquine + Primaquine 0.5 mg/kg/day for 14 days

^b^ Ordered logistic regression used to compare the proportion of patients with parasite clearance at each successive day.

Supplement Table 7. Primaquine dosing and doses completed

|  | Primaquine (7 days) | Primaquine (14 days) | *p*-value |
| --- | --- | --- | --- |
| Patients who did not completed treatment, *n* (%) | 8/325 (2.5%) | 10/329 (3.0%) | 0.651 |
| Incomplete treatment due to an adverse event, *n* (%) | 4/8 (50%) | 1/10 (10%) | 0.118 |
| Total number of doses vomited and readministered | 2/325 (0.6%) | 3/329 (0.9%) | 1.00 |
| Mean primaquine dose^a^ received, mg/kg (SD) | 0.997 (0.064) | 0.502 (0.067) | NA |

^a^ Weight based tables were used for drug dosing.

Supplement table 8. Median (IQR, range) methemoglobin measurements and adverse events during primaquine administration

| Day | CP7^a^ | CP14^a^ | DP7^a^ | DP14^a^ | p value^b^ |
| --- | --- | --- | --- | --- | --- |
| Day 0 | 0.8 (0.3 to 1, 0 to 8.7) | 0.8 (0.4 to 1.1, 0 to 7) | 0.8 (0.5 to 1, 0 to 11.4) | 0.8 (0.4 to 1, 0 to 4.2) | 0.724 |
| Day 3 | 4.2 (1.9 to 6.2, 0 to 12.1) | 2.6 (1.6 to 4.4, 0 to 13.3) | 4.4 (2.6 to 6.4, 0 to 13.7) | 2.4 (1.5 to 3.9, 0 to 10.9) | p<0.001 |
| Day 6 | 7 (4.6 to 10, 0 to 17) | 5.6 (3.4 to 7.7, 0 to 14.4) | 7.7 (5 to 9.5, 0 to 20.9) | 4.4 (2.2 to 6.6, 0 to 17.9) | p<0.001 |
| Day 10 | NA | 6.1 (4.4 to 8.4, 0 to 15.3) | NA | 5.8 (3.2 to 7.5, 0 to 15.5) | 0.276 |
| Day 13 | 3.8 (2.4 to 5.7, 0 to 12.7) | 5.7 (3.7 to 7.9, 0 to 14.5) | 3.9 (2.4 to 5.8, 0 to 11.8) | 5.3 (3.4 to 7.1, 0 to 14.3) | p<0.001 |
| Patients with peri-oral cyanosis^c^, n (%) | 8 (47) | 2 (12) | 6 (35) | 1 (6) | NA |
| Study treatment stopped^d^, n (%) | 1 (6) | 0 | 2 (12) | 1 (6) | NA |

Reported values are median (IQR, range)

^a^ CP7: Chloroquine + Primaquine 1 mg/kg/day for 7 days,

CP14: Chloroquine + Primaquine 0.5 mg/kg/day for 14 days,

DP7: Dihydroartemisinin- piperaquine + Primaquine 1 mg/kg/day for 7 days,

DP14: Dihydroartemisinin-piperaquine + Primaquine 0.5 mg/kg/day for 14 days

^b^ Non-parametric medians test used to compare differences between groups

^c^ Of the 17 patients with peri-oral cyanosis, 11 were admitted to the hospital for observation. The denominator for the percentage is the total number of patients with peri-oral cyanosis.

^d^ All patients who stopped study treatment were taking the 7-day primaquine regimen. Three of four patients had a peak methaemoglobin level <15%; one had a peak level of 20.9% on day 6 without peri-oral cyanosis and had mild dyspnea on exertion. The denominator for the percentage is the total number of patients with peri-oral cyanosis.
